# ICU-Acquired Weakness: An Unsolved Clinical Problem: A Narrative Review

**DOI:** 10.3390/jcm15103623

**Published:** 2026-05-08

**Authors:** Meghan Spoeri, Rebecca Shamberg, Nia Moragne, Richard Wlodarski, Steven B. Greenberg

**Affiliations:** 1 Department of Anesthesiology, Critical Care & Pain Medicine, Endeavor Health, Evanston, IL 60201, USA; 2 Department of Neurology, Neuromuscular Medicine, & Neurophysiology, Endeavor Health, Glenview, IL 60026, USA; 3 Department of Neurology, Pritzker School of Medicine, University of Chicago, Chicago, IL 60637, USA; 4 Department of Anesthesiology and Critical Care, Pritzker School of Medicine, University of Chicago, Chicago, IL 60637, USA

**Keywords:** intensive care unit-acquired weakness, critical illness polyneuropathy, critical illness myopathy, critical illness polyneuromyopathy, critical illness

## Abstract

Intensive care unit-acquired weakness (ICUAW) is a common and devastating complication in critically ill patients admitted to the intensive care unit (ICU). ICUAW is characterized by profound skeletal and respiratory muscle weakness and degeneration, as well as peripheral nerve dysfunction. The condition is further categorized into three primary diagnoses: critical illness myopathy (CIM), which affects skeletal muscles, critical illness polyneuropathy (CIP), which affects peripheral nerves, and critical illness polyneuromyopathy (CIPNM), which exhibits features of both CIM and CIP. Although the pathophysiology of ICUAW remains poorly understood, several risk factors have been identified, including female sex, advanced age, prolonged mechanical ventilation, extended ICU stay, prolonged immobilization, multiorgan failure, shock, infection, and other factors related to critical illness and its treatment. Currently, ICUAW is diagnosed after the onset of critical illness, and only once all other possible causes of generalized weakness have been excluded. The most commonly used assessments for suspected ICUAW are the Medical Research Council sum score (MRC-SS) and handgrip dynamometry. However, these tools require active patient participation and are, therefore, impractical for many ICU patients. Non-volitional testing methods, including electromyography (EMG) and nerve conduction studies, can be used to evaluate ICUAW, but these tests are invasive and require specialized training and resources. Due to the lack of effective diagnostic tools and an incomplete understanding of disease mechanisms, management of ICUAW is largely restricted to physical rehabilitation. ICUAW is associated with high morbidity and mortality, and survivors often experience long-term disability and reduced quality of life following hospital discharge. Future areas of research, including biomarker analysis and risk prediction modeling, may enable earlier diagnosis and intervention in critically ill patients. This review summarizes potential diagnostic tools, current management strategies, and short- and long-term prognosis and identifies emerging areas of research aimed at improving outcomes for critically ill patients with suspected ICUAW.

## 1. Introduction

Patients admitted to the intensive care unit (ICU) for severe illness or trauma often develop neuromuscular disorders characterized by severe skeletal muscle weakness, muscle degeneration, and peripheral nerve dysfunction [1]. This neuromuscular weakness is often classified as intensive care unit-acquired weakness (ICUAW) [2]. ICUAW is typically diagnosed by exclusion and defined as clinically detected muscle weakness in critically ill ICU patients for which no other cause is identified besides critical illness and its treatment [3]. ICUAW is typically generalized, symmetrical, and affects limb and respiratory muscles; facial and ocular muscles often remain unaffected [4]. The syndrome is organized into three major classifications: polyneuropathy, myopathy, or polyneuromyopathy. Critical illness polyneuropathy (CIP) stems from a neurogenic disturbance and is associated with dysfunction and deterioration of the peripheral nerves [1]. Critical illness myopathy (CIM) originates from myogenic disturbance, affecting the skeletal muscles [4]. Critical illness polyneuromyopathy (CIPNM) incorporates both CIP and CIM; the weakness originates from a combination of myogenic and neurogenic disturbances.

ICUAW is highly prevalent, affecting approximately 30–50% of critically ill patients in the ICU, with increased rates in those with sepsis, multiple organ failure, prolonged ICU stays, and extended mechanical ventilation [1,3]. The resulting weakness exacerbates these complications, leading to poorer patient outcomes. Limb and respiratory muscle weakness resulting from ICUAW commonly leads to prolonged ICU stays of greater than seven days, dependence on mechanical ventilation, and increased long-term disability [1,5]. Moreover, patients with a discharge diagnosis of CIPNM experienced, on average, hospital stays that were approximately six days longer than those of patients without a discharge diagnosis of CIPNM [6]. This prolonged length of stay increases in-hospital care costs, not including the substantial burden of long-term disability [7]. Despite high morbidity rates in patients with ICUAW, diagnostic and therapeutic modalities remain limited.

This review aims to examine the pathophysiology of ICUAW, potential diagnostic techniques, peripheral and respiratory muscle assessments, risk factors associated with developing the disorder, present methods for management of ICUAW in affected patients, and the short- and long-term outcomes in the impacted population. In addition, the article will investigate current gaps and progress in ICUAW prognosis and intervention.

## 2. Literature Search and Study Selection

We conducted a focused literature review of PubMed and Embase articles published between 2009 and 2026. Eligible publications included randomized controlled trials, observational studies (prospective and retrospective), secondary analyses, systematic reviews and meta-analyses, narrative reviews, and clinical practice guidelines involving adult ICU populations. Publications focusing on pediatric ICU populations were excluded. Given the narrative nature of this review, study selection was based on clinical relevance to ICUAW, including its pathophysiology, diagnostic approaches, risk factors, management, and outcomes. Table 1 summarizes characteristics of the included studies.

## 3. Exclusion of Alternative Causes of Weakness

ICUAW is primarily a diagnosis of exclusion and should be considered only when no other plausible cause of weakness is identified [8]. Several acute neuromuscular disorders may present with generalized weakness in critically ill patients, including Guillain–Barré syndrome (GBS), myasthenia gravis, acute inflammatory myopathies, and acute rhabdomyolysis. Among these, GBS is the most commonly encountered acute polyneuropathy in the ICU; as such, this and alternative diagnoses should be ruled out before ICUAW is suggested [9].

## 4. Pathophysiology

The pathophysiology of ICUAW is not entirely understood due to the practical and ethical challenges of studying its mechanism in humans. Current evidence suggests it may result from a combination of microvascular, neuroendocrine, bioenergetic, structural, and cellular disturbances within muscle and nerve tissue [1,4,10].

Increased permeability of endoneurial vessels can cause nerve edema and subsequent axonal injury, while critical illness triggers a catabolic hormonal state characterized by growth hormone and insulin resistance, promoting muscle breakdown [1,4,10]. Oxidative stress and insufficient clearance of damaged mitochondria and proteins may further exacerbate muscle weakness [1,10]. Structural alterations, including loss of myosin relative to actin and infiltration of fat and fibrous tissue, reduce the muscle’s ability to generate force [11,12]. In addition, membrane and ion channel dysfunction, particularly sodium channel inactivation and altered calcium homeostasis, contribute to rapid hypoexcitability and impaired contractility [1,4]. Finally, increased proteolysis and apoptosis, along with diversion of amino acids from muscles to vital organs, accelerate muscle wasting in patients with ICUAW [1,4,10,11,12].

## 5. Diagnosis

In 2014, the American Thoracic Society developed diagnostic recommendations for ICUAW [13]. Despite the availability of multiple potential diagnostic techniques, ICUAW remains difficult to identify because its clinical features overlap with other causes of weakness, and many critically ill patients cannot participate in reliable volitional muscle strength testing [8,14]. The committee emphasized that, although ICUAW is associated with adverse outcomes such as prolonged mechanical ventilation, evidence that diagnostic testing improves patient outcomes is limited. Thus, the committee recommended further studies to determine whether routine diagnosis is necessary and beneficial [13]. To date, the 2014 American Thoracic Society recommendations remain the most current ICUAW-specific clinical practice guidelines. Nevertheless, a range of potential diagnostic tools has been proposed to support the evaluation of patients in the ICU.

### 5.1. Volitional Functional Testing

Volitional functional testing assesses a patient’s strength based on voluntary effort. Two volitional bedside measures used for ICUAW assessment include the Medical Research Council sum score (MRC-SS) and handgrip dynamometry [2,3,4,15].

#### 5.1.1. Medical Research Council Sum Score

The 6-grade MRC-SS is the most widely used volitional technique for assessing ICUAW [4]. It evaluates muscle strength across twelve muscle groups, and each is graded from 0 (no contraction) to 5 (normal muscle strength). The total score ranges from 0 to 60, with scores below 48 commonly used to indicate clinically significant weakness consistent with ICUAW [2,3,4,15]. Inter-observer reliability has been reported as moderate to substantial across observational studies (κ = 0.60–0.76), suggesting reasonable reproducibility between examiners, despite some variability [15].

A modified 4-grade MRC-SS has also been developed, using four categories ranging from 0 (paralysis) to 3 (normal muscle strength) [4,16]. This simplified scale has demonstrated excellent inter-observer reliability, as well as high sensitivity and specificity when compared with the full MRC-SS. Because it requires fewer categories to assess, the scale provides clearer category distinctions and a more straightforward bedside application [16].

Despite being the most commonly used tool, the MRC-SS has limitations, as it cannot identify the underlying cause of weakness or distinguish between CIP and CIM [2,15].

#### 5.1.2. Handgrip Dynamometry and Other Functional Assessments

Handgrip dynamometry measures the isometric strength of the patient’s hand. ICUAW can generally be ruled out when grip strength is ≥11 kg in males or ≥7 kg in females [2,3,4,15]. It is a quick screening test that can help exclude ICUAW when grip strength is normal, but abnormal results typically should be followed by the MRC-SS [3]. A 2015 prospective observational study by Parry et al. examined the validity of handgrip dynamometry using these cutoff values. The study reported a sensitivity of 88% and specificity of 80% for diagnosing ICUAW when compared with MRC-based classification, which served as the reference standard. However, the relatively small sample size (n = 60) and single-center design may limit the generalizability of the findings [17].

Other physical function assessments are routinely used in the ICU but have not been specifically validated for ICUAW. Tools such as the Functional Status Score for the ICU (FSS-ICU), the ICU Mobility Scale, and the Activity Measure for Post-Acute Care are commonly used by physical therapists to evaluate mobility, monitor functional recovery, and inform discharge planning [18]. However, these instruments were developed to assess overall functional performance rather than neuromuscular weakness, limiting their applicability to ICUAW evaluation.

As with most volitional tests, handgrip dynamometry and the MRC-SS have limited practicality in the ICU, as many patients are sedated, delirious, or otherwise unable to participate in active testing. Thus, additional non-volitional tools are often used to support clinical suspicion of ICUAW and exclude alternative etiologies.

#### 5.1.3. Volitional Assessment of Respiratory Muscles

Respiratory muscle weakness occurs in approximately 80% of patients with ICUAW [2]. Volitional tests such as maximal inspiratory and expiratory pressures and maximal transdiaphragmatic pressure (Pdimax) can evaluate global respiratory muscle strength [4]. However, these assessments are not routinely used in the ICU and are not specific for diagnosing ICUAW. Although they can be informative, these measurements reflect respiratory muscle performance rather than the underlying neuromuscular disorder.

### 5.2. Non-Volitional Testing

While volitional testing can provide valuable information for the evaluation of ICUAW, its use is often limited in the ICU. Many patients cannot actively participate in strength testing, necessitating the use of non-volitional assessment tools [3].

#### 5.2.1. Electrophysiology

Electrophysiological assessments for ICUAW can be performed and include electromyography (EMG) and nerve conduction studies (NCS) [15]. Needle EMG records the electrical activity of muscles at rest and motor unit action potentials during voluntary muscle contraction, providing insight into neuromuscular function.

**Figure 1:**
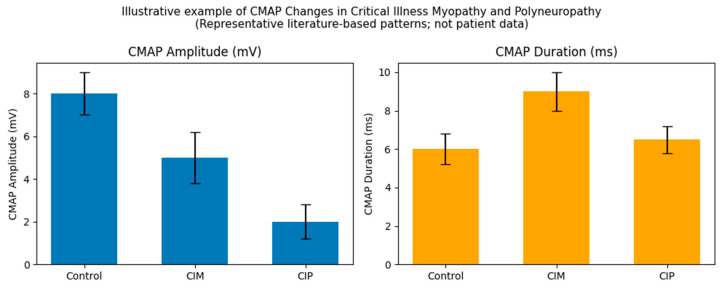
Illustrative compound muscle action potential (CMAP) amplitude and duration in controls, critical illness myopathy (CIM), and critical illness polyneuropathy (CIP). This is a representative graph; it does not depict actual patient data [22,23,24].

Nerve conduction studies evaluate both motor and sensory nerves and can generally be performed without patient cooperation. Both CIP and CIM are characterized by reduced compound muscle action potential (CMAP) amplitudes, but they differ in other electrophysiological features [2,3,4,15,19]. As a result, motor nerve conduction features may help distinguish between the two conditions. In CIM, CMAP duration is typically prolonged, whereas in CIP, CMAP duration generally remains normal. The primary differentiator is that sensory nerve action potential (SNAP) amplitudes are preserved in CIM but reduced in CIP [3,4,15,19]. Figure 1 depicts representative differences in CMAP amplitude and duration in CIM, CIP, and controls. The figure is intended for illustrative purposes only, showing what it might look like in a patient; it does not use real clinical data. For needle EMG in the evaluation of CIM/CIP, proximal muscles in both the upper and lower limbs are generally prioritized. This includes the deltoid, biceps, triceps, and vastus medialis/tibialis anterior, respectively. Some distal muscles (i.e., abductor digiti minimi) may also be assessed using needle EMG. In contrast, NCS primarily target distal muscles, namely, the abductor digiti minimi and abductor pollicis brevis in the upper extremity, and the tibialis anterior, extensor digitorum brevis, and abductor hallucis in the lower extremity.

Single nerve conduction studies, rather than comprehensive NCS, have been proposed for electrodiagnostic testing in the ICU [20]. This approach typically involves stimulation of the peroneal nerve, offering excellent sensitivity and specificity for detecting ICUAW. A 2018 prospective cohort study of mechanically ventilated ICU patients (n = 95) reported that peroneal motor NCS had a sensitivity of 94% and specificity of 91% for detecting CIPNM compared with a reference standard electrophysiological definition. However, the study was at risk of incorporation bias, as components of the index test (NCS) were included in the reference standard, potentially leading to overestimation of diagnostic accuracy [19].

Although needle EMGs and full NCS may assist in the evaluation of ICUAW, these tests are invasive (in the case of EMG), require specialized equipment and training, and may not be readily available at all hospitals [4]. However, most components of needle EMG (such as spontaneous activity) are assessed without patient cooperation. Only the assessment of motor unit recruitment and activation requires patient cooperation [1]. The limitations highlight the need for non-invasive bedside devices that not only assist in diagnosing ICUAW but also enable longitudinal monitoring of neuromuscular deterioration, even when patients cannot participate in volitional testing [21].

#### 5.2.2. Biopsy Analyses

Muscle biopsy is another potential technique used to evaluate ICUAW. Proposed diagnostic criteria for CIM include muscle biopsy findings combined with clinical and electrophysiological criteria [25]. Because bedside clinical assessments cannot reliably distinguish CIM from CIP, differentiation typically relies on electrophysiological testing, with muscle biopsy reserved for select cases and therefore rarely performed in critically ill patients [15].

A fine-needle muscle biopsy, which is less invasive than an open muscle biopsy, can be used to measure the myosin-to-actin ratio, with values below 1.7 considered abnormal [25]. Histological examination typically demonstrates muscle fiber atrophy with a preferential loss of myosin relative to actin, as well as evidence of muscle necrosis [26]. Because selective myosin loss is specific to CIM, muscle biopsy can help differentiate CIM from CIP and rule out other causes of neuromuscular weakness, particularly when other potential diagnostic measures are inconclusive [27].

Although nerve biopsy can assess axonal degeneration and myelination, and in theory, aid in distinguishing CIP from CIM, it is rarely performed in critically ill patients. In clinical practice, nerve biopsy is typically reserved for cases in which an alternative neuropathic etiology, such as suspected demyelinating neuropathy suggested by EMG, is being considered. Biopsy-based approaches, in general, are limited by their invasive nature and associated procedural risks [26].

### 5.3. Differentiating Features of CIP vs. CIM via Electrophysiological and Biopsy Studies

Distinguishing between CIP, CIM, and CIPNM is essential for accurate diagnosis and management, as the conditions may differ in pathophysiology and prognosis. Electrophysiological and muscle biopsy studies are the primary tools for differentiating CIP from CIM. Table 2 depicts key diagnostic features of CIP vs. CIM.

The diagnostic features of CIP and CIM are still being elucidated and overlap with other neuropathies and myopathies. The literature describing CIPNM is even more limited and reflects overlapping electrophysical features of both CIP and CIM. Current knowledge suggests CIPNM is typically characterized by reduced CMAP and SNAP amplitudes [19].

### 5.4. Imaging

#### 5.4.1. Ultrasound

Muscle ultrasonography (ultrasound) has emerged as a non-invasive tool to visualize muscle structure and assess changes over time in critically ill patients. This technique enables evaluation of limb muscle mass and structural alterations without requiring patient cooperation [3].

A 2022 exploratory, single-center observational study by Paolo et al. (n = 50 mechanically ventilated patients) reported a significant decrease in rectus femoris pennation angle over seven days in patients who later developed ICUAW, as assessed using the MRC-SS. These findings suggest that pennation angle may serve as an early ultrasound-based predictor of ICUAW. However, the study was limited by its exploratory design and lack of external validation [28].

A 2024 prospective cohort study of 76 mechanically ventilated patients evaluated rectus femoris cross-sectional area (RFCSA) and its atrophy rate using ultrasound. ICUAW, defined as MRC-SS < 48, was identified with a sensitivity of 76.5% and a specificity of 92.2% using a 6.9% RFCSA atrophy rate cutoff. Patients diagnosed with ICUAW based on RFCSA atrophy rate also demonstrated longer ICU stays and prolonged duration of mechanical ventilation. A key limitation of this study, however, was that strength assessments were often delayed in ICUAW patients compared to the timing of ultrasound measurements, which may have exaggerated differences between ICUAW and non-ICUAW groups [29].

Both studies are further limited by their single-center design and modest sample sizes, highlighting the need for validation in larger multicenter cohorts.

A 2025 prospective observational multicenter study of 116 septic ICU patients developed a nomogram model for predicting ICUAW. The model incorporated vastus intermedius diameter, RFCSA, and inflammatory markers, including IL-6 and C-Reactive Protein (CRP). Significant differences were observed between ICUAW and non-ICUAW groups in RFCSA, rectus femoris muscle thickness, and vastus intermedius muscle thickness, with ICUAW classification based on MRC scores. The model demonstrated high diagnostic performance, with an Area Under the Receiver Operating Characteristic Curve (AUC) of 0.966, a sensitivity of 88%, and a specificity of 95.8%. However, despite its multicenter design, the lack of external validation may limit the generalizability of these findings across broader populations and geographic regions [30].

Overall, these findings suggest muscle ultrasound may serve as a practical and objective tool for evaluating ICUAW, particularly through measurements of specific muscles, such as the rectus femoris [28,29,30]. Nevertheless, larger studies are required to confirm these findings, and standardized training will be necessary before this technology is widely adopted in critical care.

#### 5.4.2. Computed Tomography and Magnetic Resonance Imaging

Other potential adjunctive imaging-based tools include computed tomography (CT) and magnetic resonance imaging (MRI). CT-based assessments can quantify skeletal muscle mass, with one approach using the skeletal muscle index to predict ICUAW onset. A 2019 retrospective study of 23 septic patients found that low skeletal muscle index at ICU admission was associated with the subsequent development of ICUAW. However, these findings should be interpreted with caution, as the study was retrospective, single-center, and included a very small sample size [31].

MRI is another technique for muscle imaging, but its application in diagnosing ICUAW remains limited. A 2023 single-center prospective cohort study (n = 25) evaluated shoulder girdle MRI findings in patients with COVID-19-related ICUAW after ICU discharge. The study reported that edema-like peripheral signal intensities on MRI were associated with early upper proximal muscular weakness. These findings suggest a possible role for MRI in distinguishing CIM from other diagnoses. Nonetheless, a key limitation of the study was that only patients with ICUAW identified after ICU discharge (using MRC score criteria) were included, which may have resulted in an overrepresentation of more severe cases. Additionally, the MRI assessment was restricted to the shoulder girdle, limiting the generalizability of the findings to other muscle groups affected by weakness [32].

### 5.5. Summary of Potential Diagnostic Tools

Due to the lack of a “gold standard” diagnostic tool, it is challenging to determine the exact sensitivity and specificity of the measures listed in Table 3 for diagnosing ICUAW. Reported values vary across studies, influenced by differences in diagnostic thresholds, testing conditions, and the reference standards used for comparison. As a result, sensitivity and specificity estimates should be interpreted with caution, as no single test reliably identifies ICUAW in all clinical settings [15].

## 6. Risk Factors

Although ICUAW is often diagnosed after the onset of critical illness, several studies have identified risk factors for developing the condition [1,11,35].

Many risk factors for ICUAW are related to patients’ demographics and illness severity [4,30]. Common demographic risk factors are female sex and older age, as these populations typically have reduced muscle mass [33,34,35]. Additionally, higher illness severity, reflected by Acute Physiology and Chronic Health Evaluation II (APACHE II) and Sequential Organ Failure Assessment (SOFA) scores, is associated with greater ICUAW risk [36]. Factors associated with illness severity, including multiorgan failure, sepsis, infection, shock, and the need for renal replacement therapy, also increase the risk of developing ICUAW [2,29,36,37,38,39].

Other risk factors for ICUAW are related to the management of critical illness. Duration of mechanical ventilation, immobilization, and ICU stay are all well-documented risk factors for ICUAW [26,38,39].

Various medications commonly administered to critically ill patients have also been linked to ICUAW development. In a 2018 secondary analysis of a randomized controlled trial involving 172 mechanically ventilated critically ill adults, Wolfe et al. reported that days of vasoactive medication use and cumulative norepinephrine dose were independently associated with the development of ICUAW [40]. However, a dose-dependent relationship was not seen with vasopressin or phenylephrine. This finding is supported by a 2024 meta-analysis of 15 prospective and retrospective cohort studies, which suggested that vasopressor administration, particularly norepinephrine, was significantly associated with ICUAW development in critically ill adults [41]. Additionally, a 2020 meta-analysis of 10 prospective cohort studies involving 1363 critically ill adults examined the role of aminoglycoside administration in ICUAW and reported a significant relationship between aminoglycoside use and ICUAW incidence [42]. However, unlike norepinephrine, this analysis did not find duration of aminoglycoside administration to be a risk factor for ICUAW. Similarly, in a 2022 meta-analysis involving 12 cohort studies and 1950 patients, 856 of whom had ICUAW, Yang et al. identified aminoglycoside drug administration as a risk factor for ICUAW, though only three of the included studies discussed aminoglycosides [39]. Finally, while many studies have focused on the correlation between corticosteroids and neuromuscular blockers and ICUAW, overall data are conflicting, and thus these medications cannot be considered independent risk factors for ICUAW [27,36,38,39,43,44].

Several studies have also identified hyperglycemia as a risk factor for ICUAW [4,45]. Critical illness is associated with long-term inflammation and immobility that causes patients to experience stress-induced hyperglycemia and insulin resistance due to slowed glucose uptake by the skeletal muscle [10,45]. Multiple trials have found that hyperglycemia increases the risk of both CIP and CIM [10].

Nutrition is another important factor related to ICUAW. Malnutrition is known to occur in critically ill patients and is associated with prolonged mechanical ventilation [4,8]. A 2013 sub-analysis of the multicenter randomized controlled EPaNIC trial examined muscle biopsies harvested from 122 patients and 20 matched healthy controls. The authors found that late parenteral nutrition (PN) reduced the occurrence of ICUAW and promoted faster recovery for critically ill patients compared to early PN, potentially due to upregulated markers of autophagosome formation in the late PN group [46]. Additionally, a 2025 secondary analysis of 115 former ICU patients from the same trial observed that 5 years after ICU discharge, ICUAW survivors had abnormal RNA expression associated with decreased long-term muscle strength that was linked to early PN [37]. Other studies support these findings and suggest that early PN is associated with an increased risk of developing ICUAW, though the number of large randomized controlled trials focused on specific nutritional interventions for ICUAW is limited [10,26]. PN is also associated with an increased risk of infection and hyperglycemia, both of which are independent risk factors for ICUAW [47]. Additionally, an important consideration is that requiring PN often reflects greater illness severity compared to patients who can tolerate enteral nutrition (EN) [47]. Thus, the overall consensus is that early PN is associated with ICUAW risk.

## 7. Management

Aside from minimizing risk factors, there are few interventions available to prevent and treat ICUAW [2]. Management of ICUAW typically involves supportive care measures with an emphasis on physical rehabilitation [8].

### 7.1. Rehabilitative

The primary management of ICUAW is physical rehabilitation [48,49]. Early mobilization in the ICU decreases the incidence of ICUAW and reduces the time spent on mechanical ventilation and in the ICU [50]. Interventions that minimize sedation and reduce immobility by starting physical therapy early on have shown benefits to ICUAW patients [48].

However, concerns regarding the safety of enhanced mobilization have been raised. A 2022 randomized controlled trial involving 750 adult ICU patients undergoing mechanical ventilation found that increased early mobilization (daily physical therapy and sedation minimization) was associated with a higher incidence of adverse events, namely, cardiac arrhythmia, altered blood pressure, and oxygen desaturation compared to standard mobilization procedures [51].

In part motivated by these findings, a 2025 meta-analysis of 59 randomized controlled trials (n = 8462 adult ICU patients) evaluated enhanced mobilization strategies, which included earlier provision, increased duration or number of sessions, rigorous protocols, or additional tools [49]. It found that enhanced mobilization reduced the incidence of ICUAW and improved scores in the 6 min walk test and likelihood of discharge home without increasing the risk of adverse events. These results suggest that when appropriately implemented, enhanced mobilization can be both safe and effective for ICUAW patients.

In conjunction with early mobilization, neuromuscular electrical stimulation (NMES) has been studied in ICUAW patients as a potential therapy. NMES is commonly used in exercise training and rehabilitation settings to increase range of motion, reduce atrophy, and promote tissue healing [52]. Since NMES is a passive intervention, it is a viable option for patients who cannot engage in active mobilization [51]. Although a 2020 meta-analysis of six randomized controlled trials (n = 718) found that NMES did not cause significant differences in overall muscle strength, ICU mortality, or duration of mechanical ventilation and ICU stay, more recent randomized controlled trials have suggested benefits in functional outcome measures not evaluated in the meta-analysis [48]. A 2022 randomized controlled trial by Campos et al. of 139 mechanically ventilated ICU patients found that early NMES in addition to early mobilization led to better functional outcomes measured by FSS-ICU scores compared to patients who received early mobilization alone [52]. Their study also found that the NMES group had shorter hospital stay length and lower rates of ICUAW, and no adverse events were reported, suggesting NMES could be a safe preventative measure for ICUAW [52]. Additionally, a 2025 randomized controlled trial by Yokobatake et al. recently studied the effects of NMES on the muscle strength of 32 ICU patients aged 65 and older [53]. They suggested that NMES combined with early mobilization prevented lower limb weakness and improved functional outcomes including the 6 min walk test, Barthel index, and frailty status in critically ill older adults compared to early mobilization alone. Furthermore, a 2023 randomized controlled trial of 118 mechanically ventilated ICU patients suggested that NMES in conjunction with standard physical therapy led to a decrease in duration of mechanical ventilation, ICU stay, number of weaning trial failures, and ICU mortality compared to sham NMES and conventional physical therapy [54].

The discrepancy between earlier meta-analysis findings and more recent randomized controlled trials is likely due to differences in study endpoints, patient populations, and intervention protocols. While the 2020 meta-analysis focused on global outcomes, such as muscle strength, mortality, and hospital length of stay, more recent trials have shifted to focus on functional recovery measures where the benefits of NMES may be clearer. Overall, randomized controlled trials published in the past five years demonstrate significant benefits of NMES in critically ill adult patients, including improvements in functional outcomes, as well as reductions in modifiable risk factors, such as duration of mechanical ventilation and hospital length of stay. However, these trials were all single-center with relatively small sample sizes, which limits their generalizability. Thus, larger, multicenter trials that evaluate the impact of NMES on both short-term and long-term functional outcomes in ICUAW patients are needed.

### 7.2. Metabolic

Additionally, a limited number of therapeutic strategies for ICUAW focus on nutritional interventions and insulin therapy. Prioritizing EN for as long as possible is recommended for critically ill patients who are at risk of developing ICUAW [1]. In a 2020 randomized controlled trial involving 121 mechanically ventilated adults with multiorgan failure, McNelly et al. found that patients who underwent intermittent enteral feeding were better able to achieve nutritional targets than patients who experienced continuous enteral feeding, although both groups experienced similar rates of muscle loss [55]. However, patients were only studied for 10 days, which limits the generalizability of these results. Additionally, while EN is associated with more positive outcomes compared to PN, it does not prevent ICUAW from progressing [10]. Further studies on specific nutritional interventions for ICUAW management, particularly for patients who are admitted to the ICU for extended periods, are warranted.

Several trials have studied the effects of intensive insulin therapy (IIT) on ICUAW patient outcomes [10]. These studies suggested that IIT decreased overall mortality and morbidity as well as the proportion of ICU patients who developed ICUAW based on EMG and nerve conduction studies [10]. However, IIT also poses a high risk of hypoglycemia, which has been shown to increase mortality among critically ill patients [45,56]. Thus, it is recommended that IIT therapy only be used in settings where there is protection against hypoglycemia, such as continuous glucose monitoring [56]. In a 2014 secondary analysis of 104 mechanically ventilated patients previously enrolled in a randomized controlled trial focused on rehabilitation, Patel et al. demonstrated that early mobilization significantly reduced patients’ insulin requirements while still allowing them to reach the same glycemic goals as control patients [45]. This suggests rehabilitation may offer a safer approach to managing critical illness-induced hyperglycemia and insulin resistance than IIT.

### 7.3. Pharmacologic

Currently, no pharmacologic interventions exist to prevent or treat ICUAW [56]. A 1997 study suggested that IgM-enriched immunoglobulin (IVIG) therapy could prevent ICUAW [56]. However, a 2013 randomized controlled trial by Brunner et al. more recently studied the ability of IVIG to prevent ICUAW in 38 sepsis patients with early signs of ICUAW and found no effect [57]. Similar conclusions were drawn for the use of immune-modulating nutrients, specifically glutamine and antioxidants [56,58]. Although some meta-analyses identify positive effects of glutamine and antioxidant supplementation in critically ill patients, these findings are largely based on older, single-center studies with small sample sizes [58]. A 2015 review of larger, more recent trials, including the Scandinavian glutamine study (n = 400), SIGNET study (n = 502), REDOXS trial (1223), and MetaPlus study (n = 301), reported that supplementation with immune-modulating nutrients is not associated with benefits to critically ill patients as once thought, and in both the REDOXS and MetaPlus trials, these treatments even increased mortality [56,58]. Based on these findings, administration of glutamine and antioxidants is no longer a recommended intervention for mechanically ventilated patients with suspected ICUAW [56,58]. Taken together, current evidence does not support the use of pharmacologic immune-modulating interventions for ICUAW, underscoring the need for further research into novel pharmacologic approaches.

At present, the only pharmacologic-based management strategy for ICUAW is limiting administration of medications that increase risk, mainly sedatives, aminoglycosides, and vasoactive medications [8,39,40,51,59]. Specifically, norepinephrine administration should be minimized due to its dose-dependent association with ICUAW [40]. However, since these medications are essential for treating conditions related to ICUAW, such as sepsis and shock, it is often not feasible to avoid their administration to critically ill patients [41]. Thus, it is important to identify patients who require these medications as at risk for ICUAW and mitigate other modifiable risk factors for ICUAW whenever possible [41].

## 8. Prognosis

### 8.1. In-Hospital Outcomes

ICUAW is associated with a significant increase in acute morbidity and mortality [4,60]. An important consideration is that ICUAW is often related to other ICU complications, such as infection, which also increases mortality [61]. A 2014 cohort study of long-stay ICU patients conducted by Hermans et al. found that patients with ICUAW were less likely to be weaned from mechanical ventilation or be discharged from the ICU and hospital and had higher hospital costs than matched patients without ICUAW [60]. Respiratory muscle weakness, a primary feature of ICUAW, is believed to be a primary contributor to these outcomes [60,62].

### 8.2. Post-Discharge Outcomes

Patients with ICUAW who survive hospital discharge often face significant long-term effects that reduce their functional abilities and quality of life [61]. A 2015 observational cohort study of 156 adult patients, 80 of whom had ICUAW, found that ICUAW is associated with higher post-ICU mortality and diminished physical functioning in survivors 6 months after ICU discharge [63]. Additionally, Hermans et al. reported an increase in 1-year mortality among patients with ICUAW, particularly for patients who had more severe and persistent weakness at ICU discharge [60]. Compared to CIM, CIP has shown more significant long-term effects, with one review reporting that CIP can cause chronic muscle denervation up to 5 years after ICU discharge [8]. CIP is also associated with persistent sensory loss in the extremities and painful hyperesthesia [8].

However, there is also evidence that patients with ICUAW are able to make significant recoveries [8,64]. A 2022 scoping review that included 588 subjects with ICUAW found that 70.3% of subjects fully recovered and that those with CIPNM had worse outcomes compared to those with only CIP or CIM. However, only subjects who survived through the initial follow-up were included, which excluded 159 early deaths and may have inflated recovery estimates. Additionally, most studies lacked intervention details, limiting the interpretation of recovery differences [64].

While there are limited studies following long-term outcomes of ICUAW, individual case studies suggest that a multimodal continuum of care for ICUAW patients who survive the ICU can help these patients manage the long-term effects of ICUAW, such as post-intensive care syndrome (PICS), and allow them to regain physical functioning and quality of life [8]. This requires close collaboration between physicians and therapists to allow critical illness survivors to manage the physical, cognitive, and psychological aspects of recovery. PICS is a umbrella term that describes new or increased physical, cognitive, and/or psychological impairments, including ICUAW, that critically ill patients develop after ICU treatment [65]. While PICS is often associated with poor outcomes, a case report of a patient who developed PICS as a result of severe weakness developed in the ICU found that the patient made a significant recovery after undergoing several months of daily physical therapy, nutritional supplements, and support from a psychologist. Designated follow-up clinics to support ICUAW survivors and their families offer a way to coordinate this transition from the hospital back into the community [8].

## 9. Current Gaps and Future Directions

Current gaps in the diagnosis and management of ICUAW contribute to its poor prognosis. Although risk factors for ICUAW are well documented, ICUAW is often a diagnosis of exclusion made after the onset of critical illness [4,35]. This delay in diagnosis makes it difficult to prevent ICUAW or implement treatment besides supportive care. Additionally, management of ICUAW mainly relies on physical rehabilitation, which varies widely depending on clinical practice and available resources [4]. Lack of shared terminology among healthcare providers also contributes to the challenges in diagnosis, management, and prevention of ICUAW [64].

Finally, there is limited data regarding the long-term impacts of CIP and CIM on ICUAW patients who survive ICU discharge [8,61,64]. This makes it difficult to adequately support patients recovering from the effects of ICUAW after leaving the hospital. There is also a need for further study on the impact of rehabilitation and nutritional interventions on functional outcomes for ICUAW patients after ICU discharge [59,64].

### 9.1. Future Directions

#### 9.1.1. Biomarker Analysis

Although current laboratory testing cannot diagnose ICUAW, several studies have correlated various biomarkers with CIP and CIM that may make this possible in the future [1]. Identifying biomarkers for ICUAW is a critical research area with potential to streamline the diagnosis process and inform prediction models for earlier detection. Unlike MRC-SS scoring, biomarkers are objective measures of physiological processes that do not require patients to be awake and cooperative [66].

Currently, plasma IL-6, a marker of inflammation, and muscle growth and differentiation factor-15 (GDF-15), a marker of muscle atrophy, have been studied as early indicators of ICUAW [1,67]. Elevated GDF-15 levels correlate with muscle atrophy and reduced 90-day survival rates in mechanically ventilated ICU patients [66]. A 2025 systematic review of 11 cohort studies involving 1,176 critically ill adults identified 10 biomarkers associated with ICUAW, with GDF-15 having the strongest clinical utility in terms of diagnosis, predictive modeling, and prognosis [66]. Elevated serum creatine kinase (CK) has also been associated with ICUAW, but the exact sensitivity, specificity, and timeline of using CK for ICUAW diagnosis are not well studied [68].

Lei et al. recently studied biomarkers in septic patients with the goal of developing a predictive model for ICUAW diagnosis and found that the inflammatory markers IL-6 and CRP were significantly increased in patients with ICUAW. However, CRP is a nonspecific marker of systemic inflammation that could be caused by many aspects of critical illness [30,69]. Additionally, a 2021 observational cohort study of 111 COVID-19 patients, 11 of whom developed ICUAW, reported that neurofilament light chain (NfL) and glial fibrillary acidic protein (GFAp) levels were significantly increased in patients with ICUAW and in patients who later developed ICUAW during their ICU stay [70]. These biomarkers both indicate neuronal injury, and this study also found that the increase in NfL and GFAp was associated with significantly lower nerve amplitudes, further leading to the possibility of future studies linking biomarkers to ICUAW diagnosis.

#### 9.1.2. Predictive Modeling and Machine Learning

To address the delay in ICUAW diagnosis, research has shifted to developing risk prediction models for critically ill patients. Yang et al. recently developed and validated an ICUAW prediction model for adult ICU patients that considers factors such as age, gender, presence of shock, length of ICU stay, and mechanical ventilation [71]. By quickly and accurately identifying which patients are at high risk for ICUAW, this type of modeling allows healthcare providers to provide interventions such as enhanced rehabilitation as early as possible [71]. A 2025 systematic review of 25 similar prediction models found that they all demonstrated strong discriminatory ability [72]. However, most existing models are based on small cohorts and rely on MRC-SS scores for ICUAW diagnosis, limiting their widespread use and practicality for patients who are sedated [71,72].

Given these limitations, there is a push to leverage artificial intelligence (AI) to expand the generalizability of ICUAW risk prediction models [72,73]. Machine learning models can integrate heterogeneous data, account for complex relationships between risk factors, and even identify new risk factors [73]. Additionally, these models can continuously improve as new correlations emerge in clinical data [73]. Zhang et al. recently developed and compared four machine learning algorithms that incorporated bedside ultrasound data to predict ICUAW without relying on MRC-SS scoring. Another model developed and validated by Nakano et al. can predict ICUAW risk for patients within 24 h of admission using readily available data from the electronic medical record, and their model is publicly available online [74].

While there are still significant hurdles to overcome before risk prediction models become widely implemented in the ICU, continued advances in data quality and algorithm development will support their safe and reliable integration into critical care medicine.

## 10. Conclusions

ICUAW impacts a large proportion of critically ill patients and is associated with mechanical ventilation, prolonged ICU stays, extended immobility, and long-term disability. Differentiating between CIM, CIP, and CIPNM remains challenging due to limited diagnostic tools, despite important differences in their pathophysiology and prognosis. Although no definitive therapies currently exist and ICUAW remains a diagnosis of exclusion, early identification may create opportunities for more effective interventions. Emerging AI-based prediction models offer the potential to diagnose ICUAW earlier than current approaches. Future studies should continue refining these tools to help improve in-hospital outcomes for ICUAW patients. Furthermore, additional investigation should focus on improving long-term functional outcomes of ICUAW survivors to improve patients’ transitions from the hospital back to the community.

## Figures and Tables

**Table 1:** Characteristics of the included literature.

Category	Study Type	Number of Studies (n)
Primary Clinical Studies	Randomized controlled trials (RCTs)	8
	Prospective cohort/observational studies	17
	Retrospective cohort/observational studies	5
Secondary Analyses	Secondary analyses of RCT (post hoc, ancillary, sub-analyses)	8
Evidence Syntheses	Systematic reviews and meta-analyses	13
	Narrative reviews	17
Guidelines	Clinical practice guidelines	2
Other Evidence	Scoping review, pilot study, short report, and round table report	4

**Table 2:** Typical features of critical illness polyneuropathy vs. critical illness myopathy [3,4,19,24].

Diagnostic Feature	CIP	CIM
CMAP amplitude	Decreased	Decreased
CMAP duration	Normal	Increased
SNAP amplitude	Decreased	Normal
Nerve conduction velocity	Normal/near normal or slightly decreased due to loss of the fastest conducting nerve fibers	Normal/near normal
EMG at rest	Fibrillation potentials/sharp waves	Fibrillation potentials/sharp waves
Motor Unit Potential (MUP)	Long duration, high amplitude, polyphasic, reduced recruitment pattern	Short duration, low amplitude, polyphasic, early recruitment pattern
Direct muscle stimulation neCMAP:mfCMAP ratio (nerve fiber to muscle fiber CMAP ratio) [28].	<0.5	>0.5 (closer to 1)
Nerve biopsy	Nerve fiber swelling, edema with axonal degeneration, no demyelination	Normal
Muscle biopsy	Denervation atrophy of Type 1 and 2 muscle fibers	Myofiber (mainly Type 2) atrophy, loss of myosin, necrosis, fatty degeneration

CMAP = compound muscle action potential; SNAP = sensory nerve action potential.

**Table 3:** Possible tools for investigating the presence/absence of ICUAW [2,4,15,17,18,30,31].

Diagnostic Tool	Type	Advantages	Limitations	Sensitivity/ Specificity	Level of Evidence	Clinical Practice Recommendation
MRC Sum Score (MRC-SS)	Manual Muscle Testing	Easy to administer; non-invasive; reliable and valid; performed at bedside	Requires patient cooperation; does not differentiate between CIM vs. CIP; inter-observer variability; subjective component	Commonly used as a reference standard [15].	Moderate	Routine use in awake, cooperative ICU patients
Handgrip Dynamometry	Manual Muscle Testing	Easy to administer; simpler than MRC-SS; non-invasive; performed at bedside; demonstrates diagnostic validity against MRC-SS [33]	Requires patient cooperation; does not differentiate between CIM vs. CIP	Sensitivity 88%; specificity 80% (prospective single-center study, n = 60) [17].	Low– Moderate	Supplemental screening tool alongside MRC-SS in awake, cooperative ICU patients
Electromyography (EMG)	Electrophysiological Testing	Potential to differentiate CIM from CIP; can rule out other diagnoses; does not require patient cooperation in most assessments	Mildly invasive; requires specialized training; the volitional activation component requires patient cooperation	Sensitivity 47.1–100%; specificity 30.8–100% * (range across heterogeneous observational studies; narrative synthesis in systematic review) [15].	Moderate	Alternative tool when volitional testing is not feasible (sedated/uncooperative ICU patients)
Nerve Conduction Studies (NCS)	Electrophysiological Testing	Non-invasive; potential to differentiate CIM from CIP; can rule out other diagnoses; does not require patient cooperation	Costly; specialized expertise needed	Sensitivity 47.1–100%; specificity 42–91% (range across heterogeneous observational studies; narrative synthesis in systematic review) [15].	Moderate	Alternative tool when volitional testing is not feasible (sedated/uncooperative ICU patients)
Ultrasound	Imaging	Non-invasive; evaluates muscle mass and diaphragm function; patient does not need to be awake	Operator-dependent; indirect measure of muscle strength; affected by confounders such as edema and depth of sedation	Sensitivity 76% (95% CI: 0.70–0.81; specificity 80% (95% CI: 0.74–0.84) (pooled estimates from meta-analysis of 10 prospective studies, n = 561 patients) [34].	Low–Moderate	Bedside monitoring tool in ICU patients (including uncooperative patients)

* Direct muscle stimulation rather than standard EMG.

## Data Availability

No new data were created or analyzed in this study.

## Abbreviations

ICU	Intensive care unit
ICUAW	Intensive care unit-acquired weakness
CIM	Critical illness myopathy
CIP	Critical illness polyneuropathy
CIPNM	Critical illness polyneuromyopathy
GBS	Guillain–Barré syndrome
MRC-SS	Medical Research Council sum score
FSS-ICU	Functional Status Score for the ICU
Pdi_max_	Maximal transdiaphragmatic pressure
EMG	Electromyography
NCS	Nerve conduction studies
CMAP	Compound muscle action potential
SNAP	Sensory nerve action potential
ADM	Abductor digiti minimi
MUP	Motor unit potential
neCMAP	Nerve-evoked compound muscle action potential
mfCMAP	Muscle fiber compound muscle action potential
RFCSA	Rectus femoris cross-sectional area
CT	Computed tomography
MRI	Magnetic resonance imaging
APACHE II	Acute Physiology and Chronic Health Evaluation II
SOFA	Sequential Organ Failure Assessment
PN	Parenteral nutrition
EN	Enteral nutrition
NMES	Neuromuscular electrical stimulation
IIT	Intensive insulin therapy
IVIG	IgM-enriched immunoglobulin
PICS	Post-intensive care syndrome
GDF-15	Muscle growth and differentiation factor-15
CK	Creatine kinase
CRP	C-reactive protein
NfL	Neurofilament light chain
GFAp	Glial fibrillary acidic protein
AI	Artificial intelligence
